# Defense of Elevated Body Weight Setpoint in Diet-Induced Obese Rats on Low Energy Diet Is Mediated by Loss of Melanocortin Sensitivity in the Paraventricular Hypothalamic Nucleus

**DOI:** 10.1371/journal.pone.0139462

**Published:** 2015-10-07

**Authors:** Dirk W. Luchtman, Melissa J. S. Chee, Barbora Doslikova, Daniel L. Marks, Vickie E. Baracos, William F. Colmers

**Affiliations:** 1 Department of Pharmacology and Neuroscience and Mental Health Institute, University of Alberta, Edmonton, Alberta, Canada; 2 Papé Family Pediatric Research Institute, Oregon Health & Science University, 3181 SW Sam Jackson Park Rd. Portland, Oregon, United States of America; 3 Department of Oncology, University of Alberta, Edmonton, Alberta, Canada; University of Texas Health Science Center at San Antonio, UNITED STATES

## Abstract

Some animals and humans fed a high-energy diet (HED) are diet-resistant (DR), remaining as lean as individuals who were naïve to HED. Other individuals become obese during HED exposure and subsequently defend the obese weight (Diet-Induced Obesity- Defenders, DIO-D) even when subsequently maintained on a low-energy diet. We hypothesized that the body weight setpoint of the DIO-D phenotype resides in the hypothalamic paraventricular nucleus (PVN), where anorexigenic melanocortins, including melanotan II (MTII), increase presynaptic GABA release, and the orexigenic neuropeptide Y (NPY) inhibits it. After prolonged return to low-energy diet, GABA inputs to PVN neurons from DIO-D rats exhibited highly attenuated responses to MTII compared with those from DR and HED-naïve rats. In DIO-D rats, melanocortin-4 receptor expression was significantly reduced in dorsomedial hypothalamus, a major source of GABA input to PVN. Unlike melanocortin responses, NPY actions in PVN of DIO-D rats were unchanged, but were reduced in neurons of the ventromedial hypothalamic nucleus; in PVN of DR rats, NPY responses were paradoxically increased. MTII-sensitivity was restored in DIO-D rats by several weeks’ refeeding with HED. The loss of melanocortin sensitivity restricted to PVN of DIO-D animals, and its restoration upon prolonged refeeding with HED suggest that their melanocortin systems retain the ability to up- and downregulate around their elevated body weight setpoint in response to longer-term changes in dietary energy density. These properties are consistent with a mechanism of body weight setpoint.

## Introduction

Excess adiposity can be caused by prolonged exposure to a high-energy diet (HED) both in humans and in animals. Individuals are either susceptible to diet-induced obesity (DIO) or resistant (DR) to it [1, 2]. This variability in diet-susceptibility has been best studied in the animal model of HED-exposed, outbred Sprague Dawley rats [1, 3], which, like humans, maintain excess weight when returned to low energy diets [4, 5]. These animals, which we designate as DIO-defenders (DIO-D), defend elevated body weight setpoints, as do obese humans [6], unlike mice, where genetically-normal strains rarely defend excess weight [7, 8]. Though irreversible changes in the homeostatic system must accompany this relentless defense of excess body weight, the underlying sites and mechanisms remain unknown [9]. DIO-D animals may thus represent a unique model to study physiological mechanisms underlying intractable obesity.

Obesity research has focused on hypothalamic mechanisms of central energy homeostasis [9, 10]. Neurons of the hypothalamic arcuate nucleus (ARC) detect peripherally- and centrally-derived, energy balance-related signals, and mediate homeostatic responses via several downstream hypothalamic targets, including the paraventricular nucleus (PVN) [11]. The PVN is a key neuroanatomical substrate in energy homeostasis [12, 13]. It receives dense projections from ARC neurons expressing either anorexigenic melanocortin peptides or orexigenic peptides like the coexpressed neuropeptide Y (NPY) and agouti gene-related peptide (AgRP) [10, 13, 14]. Activation of central melanocortin receptors, particularly in the PVN, inhibits feeding, increases energy expenditure [15, 16] while activating NPY receptors promotes opposing responses [12, 17].

Recent work has shown some PVN neurons respond directly both to NPY and to melanocortins [18]. However, endogenous melanocortins and NPY are also thought to regulate appetite and body weight by modulating synaptic GABA release onto putative neurosecretory (NS) cells in an entirely different population of cells in the medial parvocellular PVN (mpPVN), which exhibit no postsynaptic responses to either class of neuropeptide [13]. GABAergic inputs to NS neurons arise from hypothalamic nuclei such as ARC and dorsomedial nucleus (DMN) [19, 20], where receptors for melanocortins [21] and NPY [22] are expressed. In the mpPVN, melanocortins potentiate [13], while NPY agonists inhibit, GABA release [23], consistent with their opposing actions on energy balance. We hypothesized that DIO-D animals would have reduced melanocortin, or increased NPY actions at NS cell GABA inputs. Sprague-Dawley rats were fed an HED or conventional low-energy chow diet for 10 weeks, then all were maintained on low energy diet for an additional three weeks, after which we compared NPY and melanocortin effects on NS cell GABA responses in DR, DIO-D and HED-naïve controls. In DIO-D rats, melanocortin responses were attenuated with defended excess weight compared to HED-N littermates; DR rats resembled HED-N rats. Restoring HED to DIO-D rats also restored melanocortin responses, consistent with this mechanism’s involvement in determining body weight setpoint.

## Methods

### Animals and Ethics Statement

Animal care and use was in compliance with the Canadian Council on Animal Care and approved by the Animal Care and Use Committee of the University of Alberta, Edmonton (Permit Number: AUP00000371). All animal care and use was in compliance with Society for Neuroscience Policies on the use of Animals and Humans in Neuroscience Research.

### Experiment 1: inbred OP-CD Sprague Dawley rats

This experiment tested the effect of HED on a homogeneous, inbred population of obesity-prone rats. OP-CD rats (Charles River, Wilmington MA, USA) were derived from selective inbreeding of DIO-D Sprague-Dawley rats [24]. OP-CD rats were either initially fed HED or remained on LED chow (HED-N). As detailed below, a proportion of each litter in this and the following experiment were kept HED-N to enable determination of the net weight gain of each HED-fed animal in comparison to its LED-fed littermates.

### Experiment 2: outbred Sprague-Dawley rats

This experiment tested the role of the melanocortin system in a population of outbred animals, some of which are susceptible to DIO-D. In this model, only a proportion of HED-fed rats developed obesity, while others were DR [1]. We therefore designated HED-exposed animals in the upper-third and lower-third of the net weight distribution of the overall population were were designated as DIO-D and DR, respectively. Animals in this study were bred in the University of Alberta colony, which were originally Sprague-Dawley rats supplied by Charles River Canada, and which is routinely re-invigorated with fresh breeding pairs from the original source. All experiments on the PVN of Sprague-Dawley rats from the Colmers laboratory have been performed on rats from this colony.

### Diet and experimental design

Rats were obtained at 4 weeks of age and habituated to the facility for one week prior to the start of HED feeding. Animals were housed in pairs, in a 12–12 light-dark cycle (22 ± 1°C). Body weight was measured weekly between 9am and 12pm. HED (4.60 kcal/g) contained 45% fat and 35.5% carbohydrates (Test Diet, 58V8, Richmond, IN). The LED chow (3.5 kcal/g) contained 13.2% fat and 62% carbohydrates (Lab Diet, 5053, Richmond, IN).

Starting at 5 weeks of age, weight-matched sibling animals from each litter were randomized to be HED-fed or remain HED-naïve; litters (usually n = 10) were followed as a group. Animals were provided *ad libitum* access to water and either HED or chow for 10 weeks. As an empirical test of obesity defense, all groups then received chow *ad libitum* for a minimum of 3 further weeks to establish whether HED-exposed animals in fact defended the excess body weight acquired on the energy-dense diet (i.e., DIO-D). The 3 week period was chosen based on similar feeding experiments in which animals removed from HED to chow took 3 weeks to stabilise their weight [25]. *Weight gain* was defined as individual final weight (g) at the end of week 13 minus weight at week 0. *Net weight gain*, was calculated (in both Experiment 1 and Experiment 2) as the individual weight gain (g) of an HED-exposed rat minus the average weight gain of its HED-naïve littermates at the end of week 13. At or after the end of week 13, electrophysiological studies were performed (below). In addition, some animals were sacrificed to determine hypothalamic melanocortin receptor mRNA expression. Finally, one group of rats (DIO-D_refed_ and DR_refed_) was returned to HED from week 14 to week 18 (i.e. after the chow feeding period that established their DIO-D status).

### Electrophysiological studies

Rats were lightly sedated with isoflurane then rapidly decapitated. Hypothalamic slices containing the PVN were prepared as previously described [13, 23, 25, 26]. Briefly, brains were rapidly removed and submerged in an ice-cold (2–4°C), carbogenated (95% O_2_/5% CO_2_) artificial cerebrospinal fluid (aCSF) slicing solution, containing (in mM): 118 NaCl, 3 KCl, 1.3 MgSO_4_, 1.5 CaCl_2_, 5 MgCl_2_ 6H_2_O, 1.4 NaH_2_PO_4_, 26 NaHCO_3_ and 10 glucose (300 mOsm/L). Coronal sections (250–300 μm) were cut using a vibrating slicer (Slicer HR-2, Sigmann Elektronik, Huffenhardt, Germany) and maintained in carbogenated aCSF bath solution containing (in mm) 124 NaCl, 3 KCl, 1.3 MgSO_4_, 2.5 CaCl_2_ 1.4 NaH_2_PO_4_, 26 NaHCO_3_, and 10 glucose at room temperature for at least 1 h before recording.

A single slice containing PVN was transferred into the holding chamber of a fixed stage Zeiss Axioskop FS or AxioExaminer A1 microscope (Carl Zeiss, Germany) then submerged in a constant flow (2–3 ml/min) of bath solution (34±1°C). Patch pipettes (5–6 MΩ) had an internal solution containing (in mM) 139 K-gluconate, 10 KCl, 10 HEPES, 1 EGTA, 5 MgATP, 0.3 NaGTP, 1 MgCl_2,_ 0.3 CaCl_2,_ (pH 7.25–7.30, 284–290 mOsm/L). Pipettes were connected to the headstage of an Axoclamp 2A or Multiclamp 700B amplifier (Molecular Devices, Sunnyvale, CA) used either in bridge current clamp or continuous single electrode voltage clamp mode. Data were filtered at 3 kHz and sampled at 5–10 kHz using a Digidata 1322A or 1440A computer interface and pClamp software (versions 8.2 or 10.2, Molecular Devices). Membrane potential values reported here were not corrected for the calculated liquid junction potential (~+14 mV).

We previously determined that the greatest sensitivity to feeding-related peptides in the PVN is in the GABA inputs to putative mpPVN neurosecretory (NS) cells [13, 27]. Neurons chosen for study fired tonically, did not burst, and were readily differentiated either from putative magnocellular or parvocellular pre-autonomic cells by previously published criteria [27–32]. Although properties of all neurons studied were consistent with those of NS cells, the original defining criteria included the projection targets of individual neurons, which we did not determine here, so we consider our neurons to be ‘putative’ NS cells [27]. The resting membrane potential (RMP) of NS cells ranged between -55 – -60 mV. Neurons were held in voltage clamp at a holding potential (V_h_) of -60 mV for most of the experiment. Neurons were only studied if their holding current at RMP and access resistance remained stable in voltage clamp for 10–15 min after establishing a whole-cell recording. Cells with changes in access resistance > 10% were excluded from analysis.

VMN neurons were selected based on their anatomical location within the oval area on either side of the third ventricle as viewed under low magnification, high-contrast illumination [33]. Electrophysiological recordings were performed as above. Changes in membrane potential were measured as deviations from RMP while measurements of rheobase were determined from cells brought to -60 mV with steady state current as needed.

Inhibitory postsynaptic currents (IPSCs) were evoked and recorded in mpPVN NS neurons as previously described [13, 23, 27]. In brief, synaptic responses were recorded upon electrical stimulation via either a sharpened tungsten monopolar electrode or a saline-filled glass patch pipette, positioned either within or just adjacent to the PVN as needed to elicit optimal synaptic responses. IPSCs were evoked by 100–200 μs, 1–20 V stimuli at V_h_ = -30 – -40 mV. Cells were returned to V_h_ = -60 mV once IPSC responses were acquired. Stimulus amplitudes were chosen to elicit responses at between 60–75% of the maximum IPSC amplitude initially established for each cell. Six successive IPSCs, evoked at 0.05 Hz were digitally averaged. Prior to any pharmacological manipulations, this protocol was repeated every 5 minutes until the responses were deemed stable. The last averaged IPSC value before drug application was used as the baseline. A -10 mV, 200 ms voltage step was applied after each stimulus to assess pipette access resistance. MTII (Phoenix Pharmaceutical, Belmont, CA or PolyPeptide Laboratories, San Diego, CA), human/rat NPY (Peptidec Technologies, Pierrefonds QC, Canada, or PolyPeptide) and bicuculline (Sigma, St. Louis, MI), were dissolved in warmed, carbogenated aCSF from a concentrated stock and applied via bath perfusion for 5 min. All drugs were applied at V_h_ = -60 mV and washed out for at least 25–40 min. To establish the time-course of a drug effect, the cell was held at the more depolarized potential at several time points for 2 min, and 6 successive IPSCs were averaged as above. Only drug effects that at least partially reversed upon washout (≥ 50%) were used for statistical analyses. No significant effects of either NPY or MTII were observed on postsynaptic properties (resting membrane potential, action potentials, input resistance, membrane current-voltage relationship) in any of the recordings from mpPVN neurons in this study, clearly differentiating this population from those MC4R-expressing PVN neurons studied in MC4R-GFP mice [18].

### *In situ* hybridization

Brains from outbred DR and DIO-D animals were frozen over dry ice, then coronal sections (20 μm) were cut on a cryostat and thaw-mounted onto Superfrost Plus slides (VWR Scientific, Radnor PA). Hypothalamic sections were collected in a 1:6 series from the suprachiasmatic nucleus (Bregma -1.0mm) caudally through the mammillary bodies (Bregma -5.00 mm). Antisense ^33^P-MC4R riboprobe corresponding to nucleotides 557–1006 of rat MC4R (0.23 pmol/ml; GenBank accession no. NM_013099.2) or ^33^P-MC3R riboprobe corresponding to nucleotides 90–486 of rat MC3R (0.22 pmol/ml; NM_001025270.3) was denatured, dissolved in hybridization buffer along with 1.7 mg/ml tRNA, and applied to slides. Slides were covered with glass coverslips, placed in a humid chamber, and incubated overnight at 55°C. The next day, slides were treated with RNase A and washed under conditions of increasing stringency. Slides were dipped in 100% ethanol, air dried, and then dipped in NTB-2 liquid emulsion (Kodak). Slides were developed after 18 days and coverslipped. All slides were counted unilaterally by an observer blinded to treatment under dark-field illumination with custom software [33] designed to count the total number of cells and the number of silver grains (corresponding to ^33^P-labeled melanocortin receptor mRNA) over each cell. Cells were not counterstained, but could be clearly distinguished with conventional phase contrast illumination [33]. Data were from 9 DR and 5 DIO animals; 4 sections were evaluated per animal, cell numbers are as indicated in the legend. Cells with a signal-to-background ratio of at least 2 or greater were considered to express receptor mRNA. Data are expressed as the total number of identifiable cells and grains/cell (a semiquantitative index of mRNA content/cell).

### Statistics

Statistical analyses were performed using Prism versions 5 or 6 (GraphPad, San Diego, CA). Body weight was analyzed by repeated measures two-way ANOVA (RM-ANOVA) and treatment effects compared by post-hoc Bonferroni tests where appropriate. For electrophysiology, RM-ANOVA with posthoc Bonferroni tests were used to analyze the time-course of drug effects on IPSCs *within* each group (HED-N, DR and DIO-D) and IPSC amplitude at each time point compared to baseline. *Between* group comparisons of peak drug effects were analyzed with independent-sample t-tests (experiment 1) or one-way ANOVA with Newman-Keuls multiple comparisons (experiment 2). *Between* group comparisons of whole hypothalamic/regional mRNA expression were measured with one-way ANOVA. The strength of the relationship between weight gain and drug effect was analyzed using the Pearson correlation co-efficient (*Pearson r*) with linear regression analyses. Electrophysiological data from up to 4 separate neurons were obtained from the PVN of a given animal, though we usually only obtained data from only one neuron per animal (the average was between 1.2 and 1.5 cells per animal, depending on the experiment). To prevent animals with data from more cells from biasing our results, we compared the relationship between weight gain and drug effect using either data points from each neuron from a given animal or instead using a single point with the average response for all neurons from that animal. There was no significant difference in the slopes of the line or the significance of the correlation between the two analyses, so we retained the individual data points in the analysis. Significance was pre-determined to be at p < 0.05. Tests of normality used to assess body weight distributions were the KS normality test, D'Agostino & Pearson omnibus normality test and Shapiro-Wilk normality test. Numbers (n) for each group are indicated in parenthesis throughout the figures.

## Results

### Experiment 1: Body weight and neuropeptide responses in OP-CD rats

#### Obesity is defended in HED-exposed OP-CD rats

**Fig 1:**
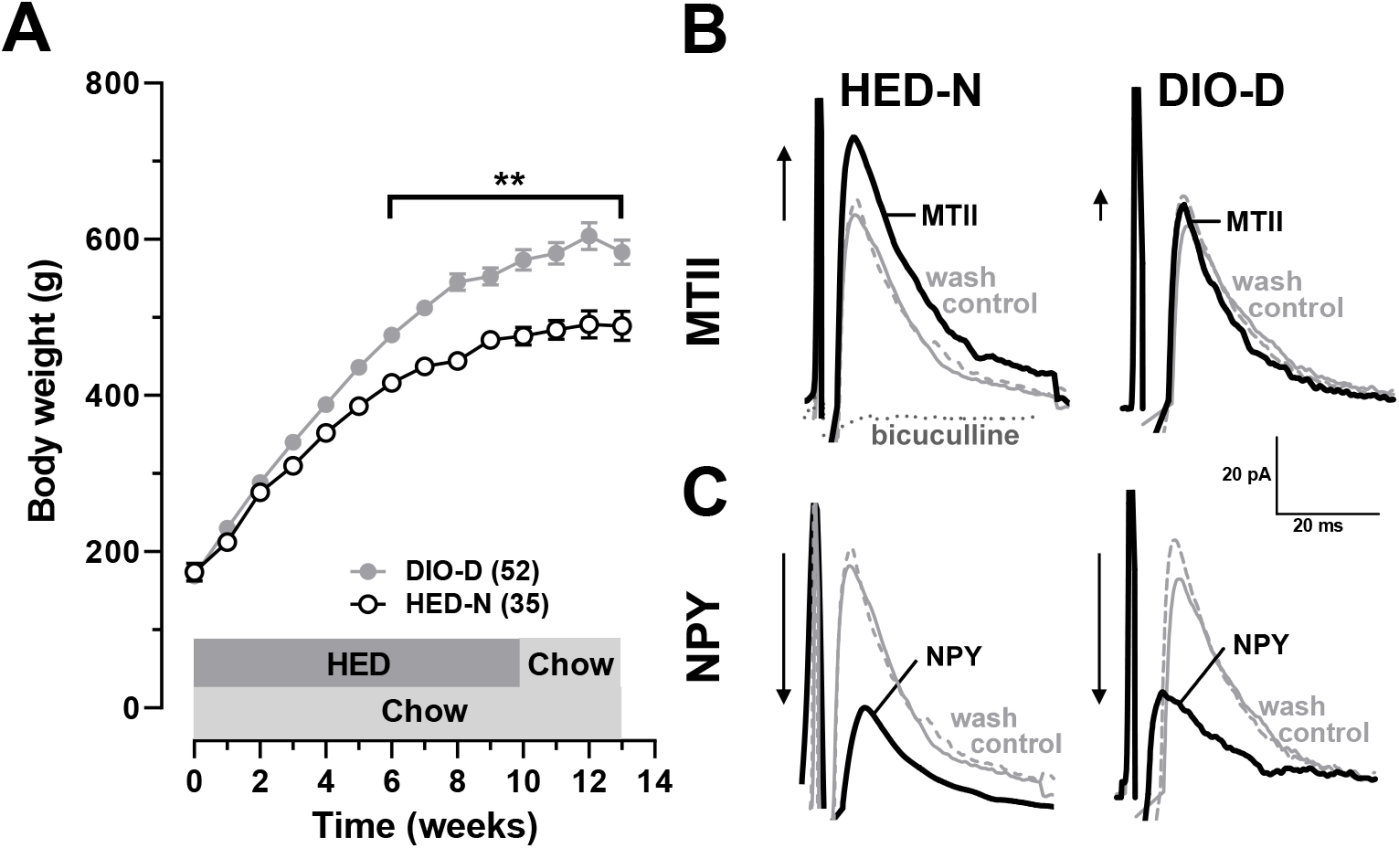
HED-fed, inbred obesity prone (OP-CD) rats have reduced responses to MTII. (**A**) Weekly mean body weights of OP-CD strain Sprague-Dawley rats fed either HED or chow. When returned to chow, HED-fed rats defended their diet-induced obesity (DIO-D) relative to HED-naïve (HED-N) littermates. (**B**) Representative IPSC recordings from neurosecretory (NS) mpPVN cells from HED-N (*left*) and DIO-D (*right*) rats superimposed in control (solid gray), 100 nM MTII (black) and after washout (dashed gray). IPSCs are abolished by application of 50 μM bicuculline (dashed dark grey, *left*). Arrows to the left of traces indicate the relative direction and magnitude of MTII-mediated changes. MTII responses were significantly attenuated in PVN NS cells of DIO-D rats relative to HED-N rats. (**C**) Representative IPSC recordings from HED-N (*left*) and DIO-D (*right*) rats superimposed in control (solid gray), 300 nM NPY (black) and wash (dashed gray). Arrows to the left of the traces indicate the relative direction and magnitude of NPY-mediated IPSC changes. There was no significant difference between the effects of NPY in PVN NS cells of DIO-D and HED-N rats. ** p < 0.01.

Five week-old OP-CD rats (n = 52) were fed HED for 10 weeks, followed by chow for 3 weeks, while weight-matched, HED-N littermates (n = 35) remained on chow throughout for comparison. After the initial 10 weeks, HED-fed animals demonstrated greater weight gain than HED-N littermates (HED-fed: 575 ± 13g; HED-N: 477 ± 11g, p < 0.01; **Fig 1A**). Final body weights in HED-fed rats remained greater than for HED-N even 3 weeks after terminating HED-feeding, thus we classified them as DIO-D (583 ± 16g vs. HED-N: 489 ± 19g, p < 0.01).

#### Melanocortin, but not NPY responses are attenuated in the PVN of DIO-D OP-CD rats

We tested whether GABA inputs to mpPVN cells were equally sensitive to the synthetic melanocortin analogue, melanotan II (MTII) and NPY in hypothalamic slices from DIO-D and HED-N OP-CD rats. We studied bicuculline (50 μM)-sensitive, GABA_A_ receptor-mediated, evoked inhibitory postsynaptic current (IPSC) responses in whole cell recordings from mpPVN NS cells (**Fig 1B**). Similar to our previous observations in naïve adult Sprague Dawley rats [13, 22, 24, 25], MTII (100 nM) reversibly facilitated IPSCs in NS cells both from HED-N and DIO-D rats (100 nM, **Fig 1B**). However, the effect of MTII in DIO-D NS cells (+16.3 ± 3.6%, n = 41) was only half of that seen in HED-N neurons (+31.6 ± 3.6%, n = 10, p < 0.001). In contrast, these evoked IPSCs were inhibited by NPY (300 nM, **Fig 1C**) to a similar extent in neurons from DIO-D (-28.3 ± 2.3%, n = 43) and HED-N NS rats (-33.1 ± 3.2%, n = 12, p > 0.1 vs DIO-D).

**Fig 2:**
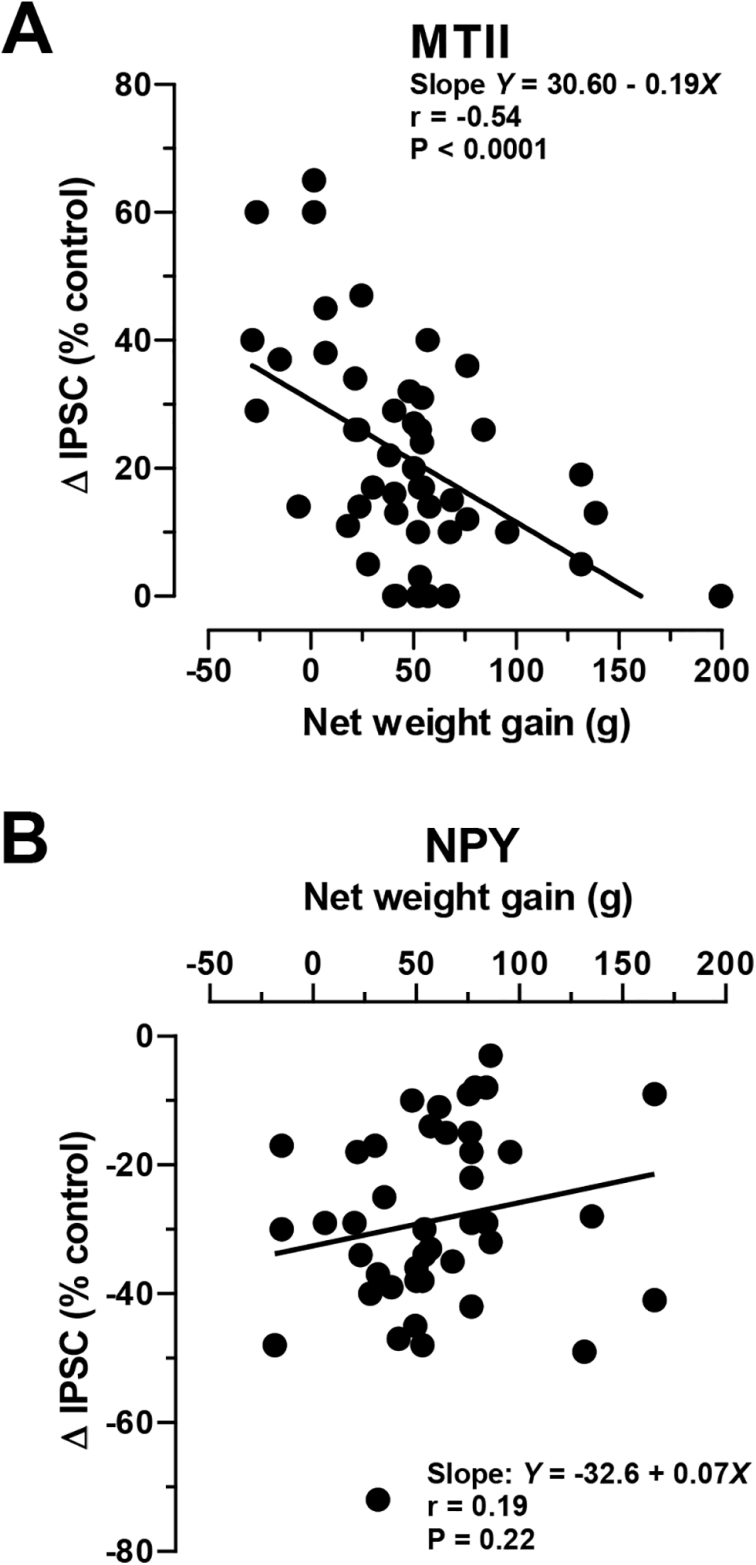
Net weight gain in inbred DIO-D OP-CD rats correlates with reduced MTII, but not NPY effects in mpPVN. Corresponding individual net weight gain plotted against either (**A**) MTII- (100 nM; n = 50 from 34 rats) or (**B**) NPY-mediated (300 nM; n = 43 from 31 rats) IPSC amplitude change in individual electrophysiological recordings from neurons from all HED-fed OP-CD Sprague Dawley rats tested. *Text insets*: linear regression analysis and Pearson r value.

We then examined whether a relationship existed between individual net weight gain and the presynaptic effects of the peptides on neurons from that animal. When we plotted the IPSC changes in response to MTII and NPY application from individual NS neurons against individual net weight gain for all HED-exposed animals (see Methods), we observed that MTII response magnitudes and net weight gain were inversely related. Thus, responses to 100 nM MTII were smaller in rats with greater net weight gain (*r = -* 0.54, p < 0.0001, **Fig 2A**). By contrast, there was no similar relationship between net weight gain and the response to 300 nM NPY (*r =* 0.19, p = 0.11, **Fig 2B**).

#### NPY responses are attenuated in VMN of HED-fed OP-CD rats

**Fig 3:**
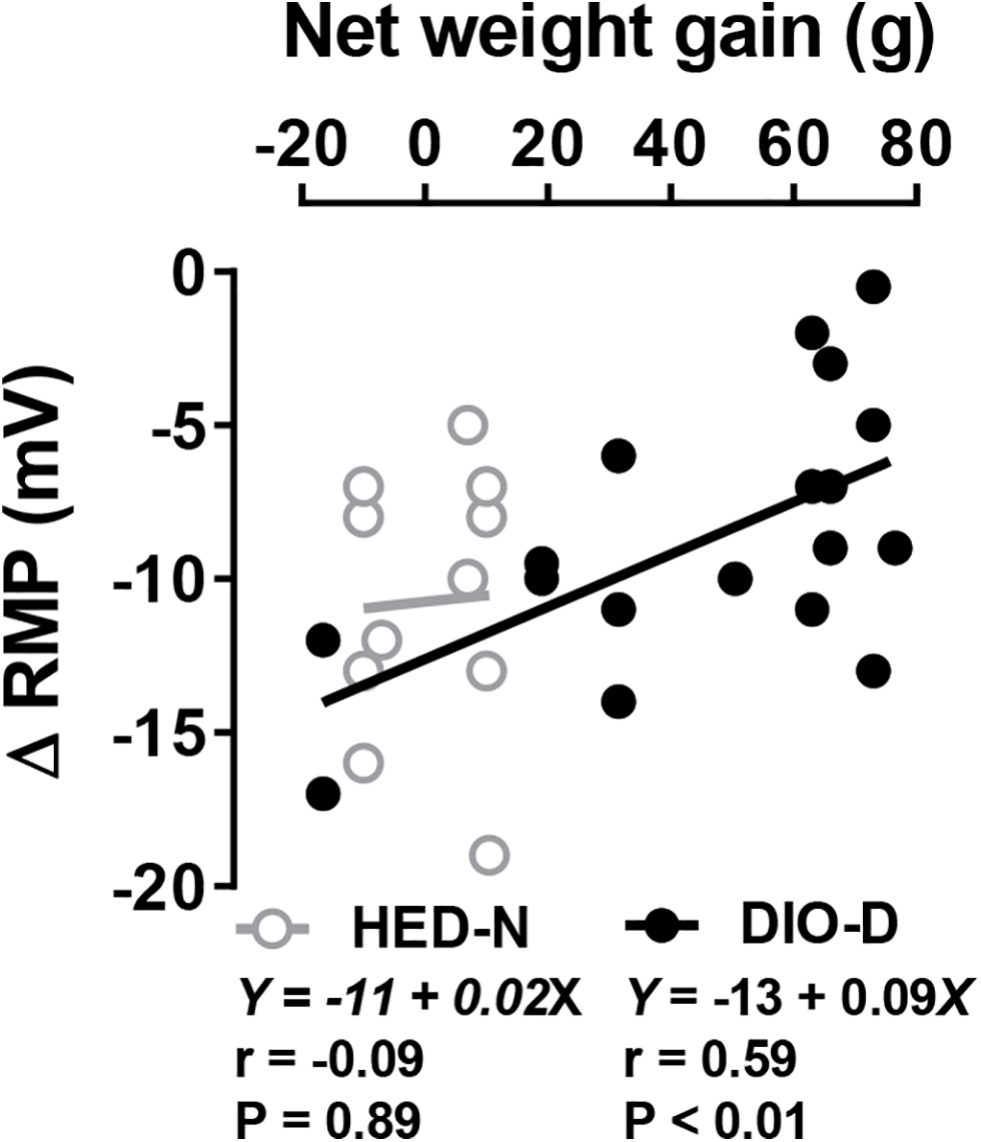
The effect of NPY is attenuated in neurons within the dorsomedial and central VMN of inbred DIO-D OP-CD rats. NPY (500 nM) caused a smaller inhibition in VMN neurons from DIO-D rats than in those from HED-N rats. Plot shows individual weight gain versus corresponding NPY-mediated hyperpolarization (ΔRMP) in individual neurons from HED-fed (black circles; n = 18 from 8 rats) and HED-N (grey circles; n = 11 from 5 rats) rats. Lines indicate slope of regression lines. *Text insert below—*linear regression and Pearson r values.

While melanocortin responses in the PVN were seen to decline with excess body weight, we considered whether NPY responses might be elevated in other hypothalamic regions to account for the defense of excess body weight in DIO-D rats returned to chow. Other centers, like the ventromedial hypothalamic nucleus (VMN) play a role in NPY-mediated energy balance [31, 33]. A subset of leptin-sensitive VMN neurons is directly inhibited by NPY [31]. We therefore determined if NPY responses are altered in VMN neurons of DIO-D animals. HED-feeding alone had no effect on the resting membrane potential (RMP; HED-N: -61.2 ± 1.0 mV, n = 7; DIO-D: -60.1 ± 0.9 mV, n = 7) or resting excitability, measured by the baseline rheobase current (HED-N: 27.4 ± 3.1pA, n = 7; DIO-D: 24.0 ± 2.9pA, n = 7; p > 0.05) of OP-CD VMN neurons. In VMN neurons from HED-N animals, NPY hyperpolarized (by -10.4 ± 0.7 mV, n = 17) and increased the rheobase (by 31.4 ± 8.3 pA, n = 17) of HED-N VMN cells, comparable to previous observations [31]. However NPY’s effects were significantly reduced in VMN cells from DIO-D animals (RMP:-7.9 ± 1.0, n = 16; p < 0.05 vs. HED-N; rheobase: 11.4 ± 3.4 pA, n = 7; p < 0.05 vs. HED-N). Moreover, this loss of NPY-sensitivity strongly correlated with net weight gain in DIO-D rats (*r =* 0.59, p < 0.01; **Fig 3**).

### Experiment 2: Body weight and neuropeptide responses in outbred SD rats

#### Obesity is defended in a subset of HED-exposed outbred rats

To better model the diversity and variability in body weight regulation in humans, we examined the effect of HED-feeding on body weight in outbred Sprague Dawley littermates (n = 150), which were weight-matched and randomized to be either HED- (10 weeks) then chow-fed (3 weeks) or chow-fed throughout (13 weeks, n = 100). We first determined the proportion of HED-fed rats that either defended (DIO-D) or resisted (DR) defending elevated body weights when returned to chow after the HED regimen. We then tested whether neurons in mpPVN of DIO-D rats have reduced responses to MTII relative to their DR or HED-N littermates.

**Fig 4:**
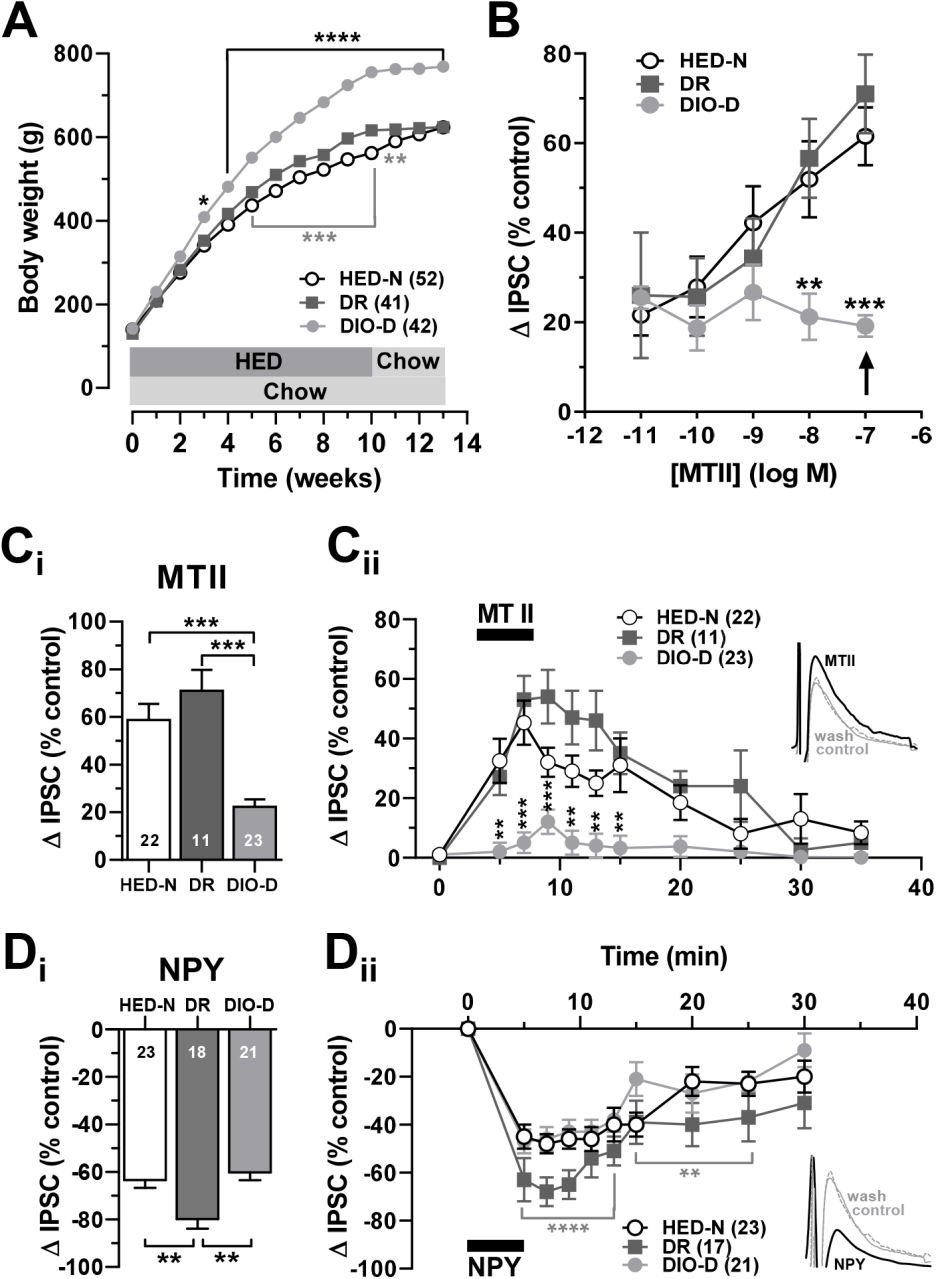
Outbred HED-fed Sprague Dawley rats that defend elevated body weights have reduced melanocortin responses. (**A**) Weekly mean body weights of outbred Sprague Dawley rats fed HED or chow. Relative to HED-N littermates, some HED-fed animals defend excess body weight (DIO-D), while some are resistant (DR) to HED-induced weight gain (Methods). Asterisks indicate significant weight differences between HED-N and DIO-D (black *) or DR (gray *), respectively, during HED-feeding. When returned to chow for 3 weeks, DR rat weights were not different from those of HED-N rats. (**B**) Concentration-response relationship of peak MTII effects on IPSCs recorded in mpPVN neurons from HED-N, DR or DIO-D rats. Asterisks indicate significant differences between MTII effects on IPSCs from DIO-D versus HED-N. *Arrow* indicates effects at 100 nM MTII. (**Ci**) Peak increase in IPSC amplitude (Δ IPSC) elicited by 100 nM MTII in NS neurons from HED-N, DR and DIO-D rats. MTII effects were not different in neurons from DR and HED-N animals. (**Cii**) Time course of 100 nM MTII’s effect on IPSC amplitudes recorded in NS neurons from HED-N, DR and DIO-D rats. Asterisks (*) indicate significant differences between IPSC responses in DIO-D versus DR or HED-N. Numbers of neurons studied are indicated in figure legend. *Inset*, superimposed representative IPSC traces illustrating the MTII effect in an HED-N neuron. (**Di**) Peak decrease in IPSC amplitude elicited by 300 nM NPY in NS neurons from HED-N, DR and DIO-D rats. NPY effects in HED-N and DIO-D animals did not differ. (**Dii**) Time course of 300 nM NPY effect on IPSC amplitudes from HED-N, DR and DIO animals. Note that NPY responses from DIO-D and HED-N rats overlap. Asterisks (gray *) indicate significant differences between IPSC responses in DR versus DIO-D or HED-N rats. * p < 0.05, ** p < 0.01, *** p < 0.001.

Net weight gain associated with HED exposure was normally distributed in the outbred rats (not illustrated). Evaluating the entire population of HED-fed rats at the end of the experiment, the most obesity-prone (upper 1/3 of net weight gained) were designated DIO-D (n = 50), while the least obesity-prone (lower 1/3, n = 50) were designated DR [1, 2, 5, 34, 35]. After returning to chow for 3 weeks, the body weight of DR rats (628 ± 4.6 g, n = 50,) was not different from HED-N littermates (623 ± 4g, n = 100; **Fig 4A**) but DIO-D rats remained substantially heavier (759 ± 6g, n = 50; p < 0.0001 vs. either DR or HED-N). In a representative cohort, DIO-D rats (139 ± 4 kcal/day, n = 5) consumed significantly more calories than did DR rats (111 ± 2 kcal/day, n = 4; p < 0.001) during the last week of the HED feeding period, and maintained this elevated caloric input (130 ± 3 kcal/day, n = 5) relative to DR rats (105 ± 8 kcal/day, n = 4; p < 0.01) even after 3 weeks on chow. These results suggest that the maintenance of caloric intake is well regulated in the DIO-D animals fed either chow or HED.

#### MTII responses are reduced in PVN NS neurons of outbred DIO-D rats

We compared IPSC responses to 100 nM MTII in mpPVN NS cells from DIO-D, DR and HED-N rats. MTII reversibly increased the IPSC amplitude to a similar amount in all DR and HED-N controls. As in Experiment 1, this effect was sharply attenuated in DIO-D rats (**Fig 4B and 4C**). The peak effect of 100 nM MTII was considerably smaller in neurons from DIO-D rats (+22.1 ± 3%, n = 23) than from DR (+71.3 ± 8%, n = 11; p < 0.001) or HED-N rats (+58.7 ± 7%, n = 22; p < 0.001, **Fig 4Ci**). MTII effects in neurons from HED-fed rats in the middle 1/3 of the net weight gain distribution were also smaller (+40.2 ± 7.7%, n = 12) than those from DR or HED-N rats, but were greater than those from DIO-D rats. We further tested NS neurons from DIO-D rats using different MTII concentrations, and showed that DIO-D neurons had sharply lower peak responses to MTII throughout the entire concentration range tested (10 pM -100 nM) when compared to responses in HED-N or DR neurons (**Fig 4B)**. In fact, GABA inputs to NS cells from DIO-D rats showed no dependence on MTII concentration in these experiments (**Fig 4B**). By contrast, neurons from the DR and HED-N rats had similar concentration-dependent responses to MTII, with EC_50_ values comparable to a previous report [13].

By contrast, NPY (300 nM) reversibly and robustly decreased the IPSC amplitude in neurons from all groups of rats (**Fig 4D**). Surprisingly, while neuronal responses to 300 nM NPY in DIO-D (-60 ± 4%, n = 23) rats did not differ significantly from those in HED-N rats (-63.4 ± 3.6%, n = 21), responses to NPY in DR rats, were significantly elevated (-79 ± 5%, n = 17; p < 0.01 vs. HED-N or DIO-D; **Fig 4D**). The timecourse of the responses to either 100 nM MTII (**Fig 4Cii**) or 300 nM NPY (**Fig 4Dii**) were similar.

**Fig 5:**
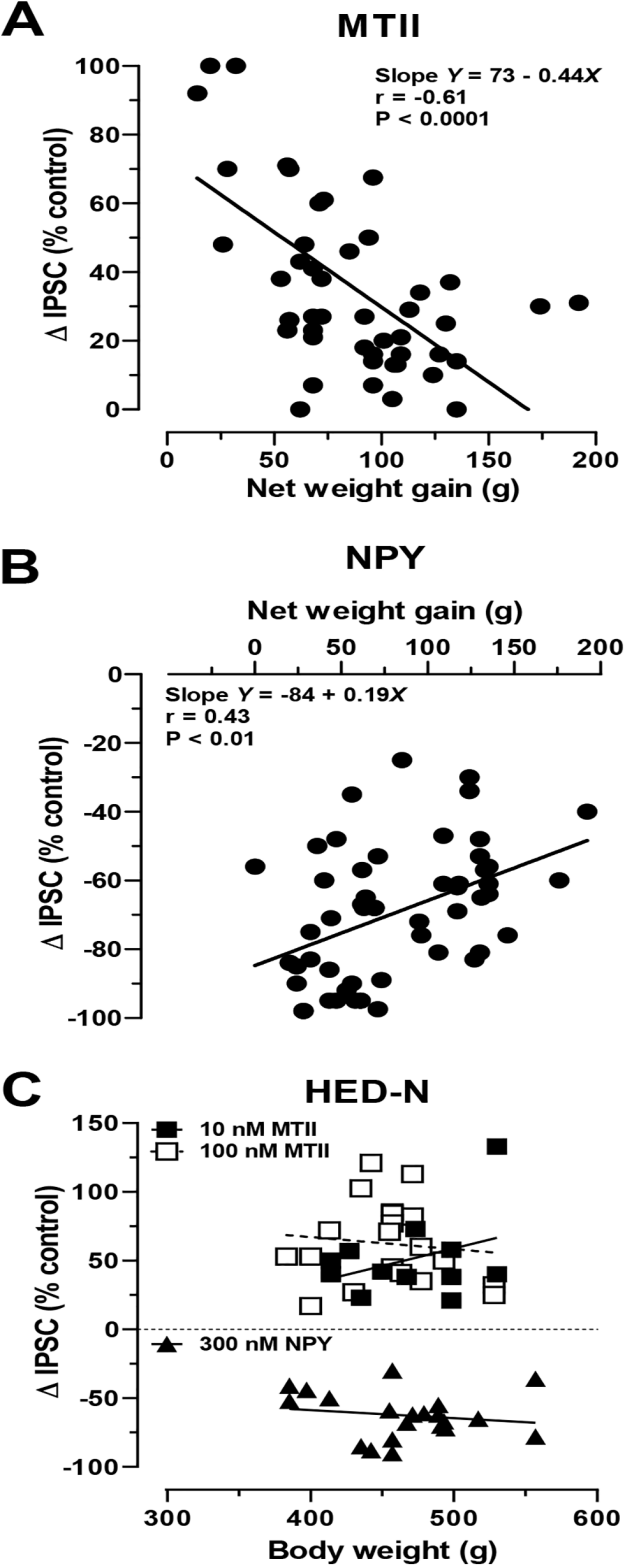
Net weight gain in outbred HED-fed Sprague Dawley rats correlates with attenuation of MTII responses, while NPY responses are paradoxically increased in DR, not DIO-D rats. Individual net weight gain versus (**A**) MTII- (100 nM; n = 47 from 34 rats) or (**B**) NPY- (300 nM; n = 51 from 36 rats) mediated changes in IPSC amplitude obtained from neurons in HED-fed (**A**, **B**) rats, or versus body weight from HED-N (**C**) rats. In C, MTII (100nM) n = 20 from 14 rats; MTII (10 nM) n = 12 from 8 rats; NPY (100nM) n = 21 from 16 rats. *Insets* in **A** and **B** show the respective linear regression analysis and Pearson r values. Overall body weight and peptide responses were not significantly correlated in NS neurons from HED-N rats.

As in Experiment 1, we plotted NS neuron IPSC responses to MTII or NPY application against individual net weight gain in neurons from all HED-exposed rats. There was a strong and significant negative correlation between net weight gained and MTII response (*r =* -0.61; p < 0.0001; **Fig 5A**). NPY responses also correlated negatively with net weight gain (*r =* 0.43; p < 0.01; **Fig 5B**), but were more weakly correlated than MTII responses, mainly reflecting the paradoxical increase in NPY-sensitivity at DR NS neurons. By contrast, the final body weights of HED-N animals did not correlate with either MTII or NPY responses (**Fig 5C**).

#### Melanocortin receptor expression is reduced in DIO-D vs. DR rats

**Fig 6:**
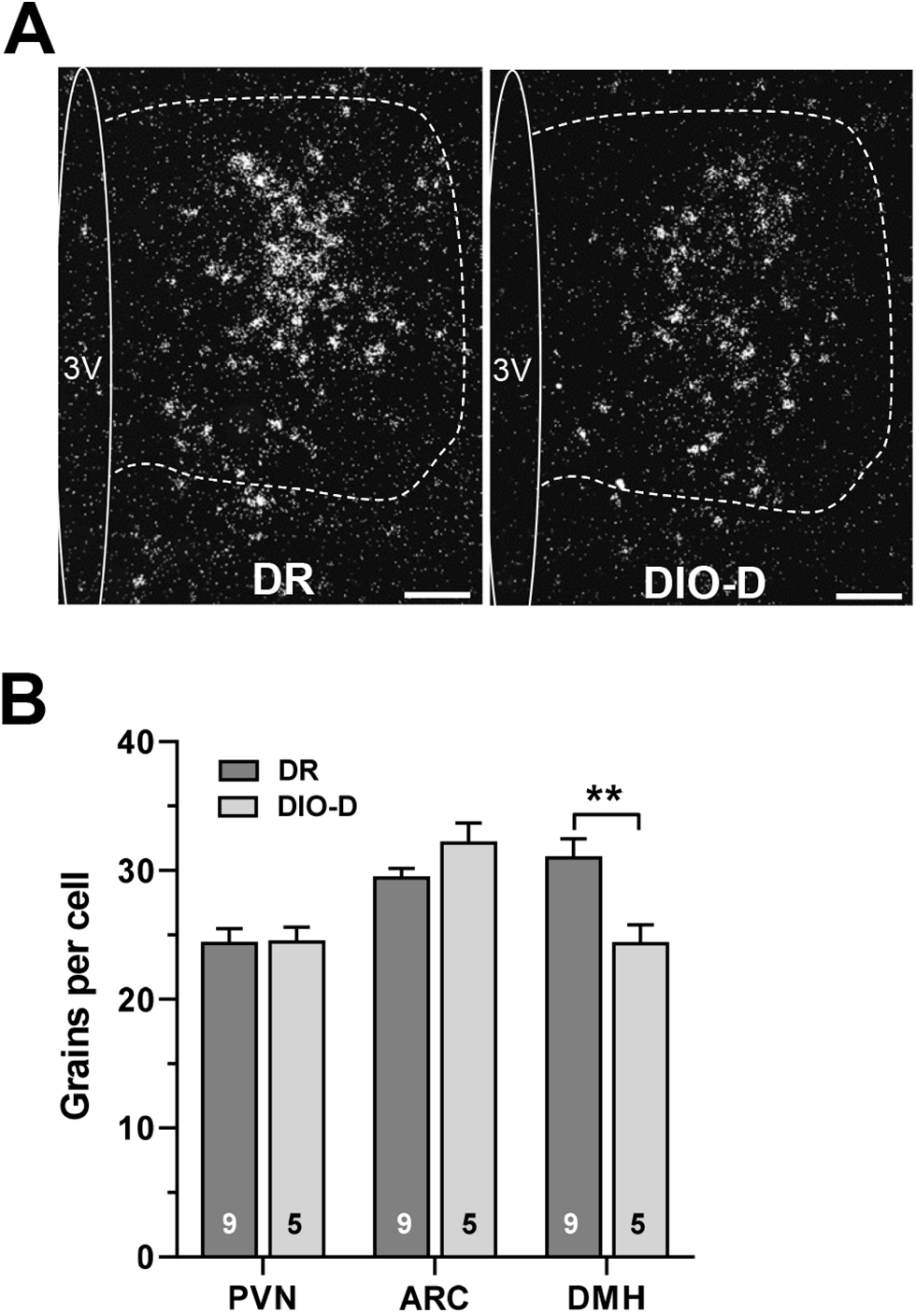
Melanocortin 4 receptor (MC4R) expression is reduced in DMN of outbred DIO-D Sprague Dawley rats. (**A**) Representative darkfield photomicrographs showing *in situ* hybridization of MC4R mRNA gene expression in the DMN (outlined region) from DR (*left*) and DIO-D (*right*) outbred rats. Scale: 100 μm. 3V, third ventricle. (**B**) In situ hybridization for MC4R resulted in fewer silver grains over individual neurons in DMN, but not PVN or ARC of DIO-D vs. DR rats. Data were obtained from 9 DR and 5 DIO rats. Numbers of cells studied per animal: PVN—n = 113 ± 15 (DR), n = 97 ± 15 (DIO), p = 0.5; ARC—n = 61 ± 4 (DR), n = 60 ± 7 (DIO), p = 0.8; DMH—n = 127 ± 13 (DR), 92 ± 14 (DIO), p = 0.12. ** p < 0.01.

Synaptic recordings in NS cells showed that HED-feeding had the greatest impact on sensitivity to the melanocortin agonist. We next examined whether HED-feeding differentially regulated the expression of melanocortin receptors in the hypothalamus of DIO-D, DR and HED-N rats. Melanocortin responses are presynaptic to NS neurons, thus we examined melanocortin receptor MC3R and MC4R expression in GABAergic hypothalamic areas known to innervate the PVN [19, 36, 37], including the peri-PVN region, ARC and DMN (**Fig 6A**). While no differences were seen between MC4R mRNA levels seen in either the peri-PVN region or arcuate of DIO-D and DR animals, MC4R expression was downregulated in the DMN of DIO-D animals (**Fig 6B**). By contrast, MC3R mRNA levels [36, 37] were either undetectable (in the periPVN and DMN) or unchanged (ARC: DIO-D 20.1 ± 1.1 vs. DR 19.3 ± 1.4). In addition, MC3R mRNA was unchanged in the VMH (DIO-D 24.8 ± 1.3 vs. DR 25.3 ± 0.8). The selective decrease of MC4R expression in the DMN is consistent with the loss of melanocortin sensitivity by GABA inputs to NS neurons in the DIO-D animals.

#### Loss of MTII response is reversed in DIO-D rats returned to HED

**Fig 7:**
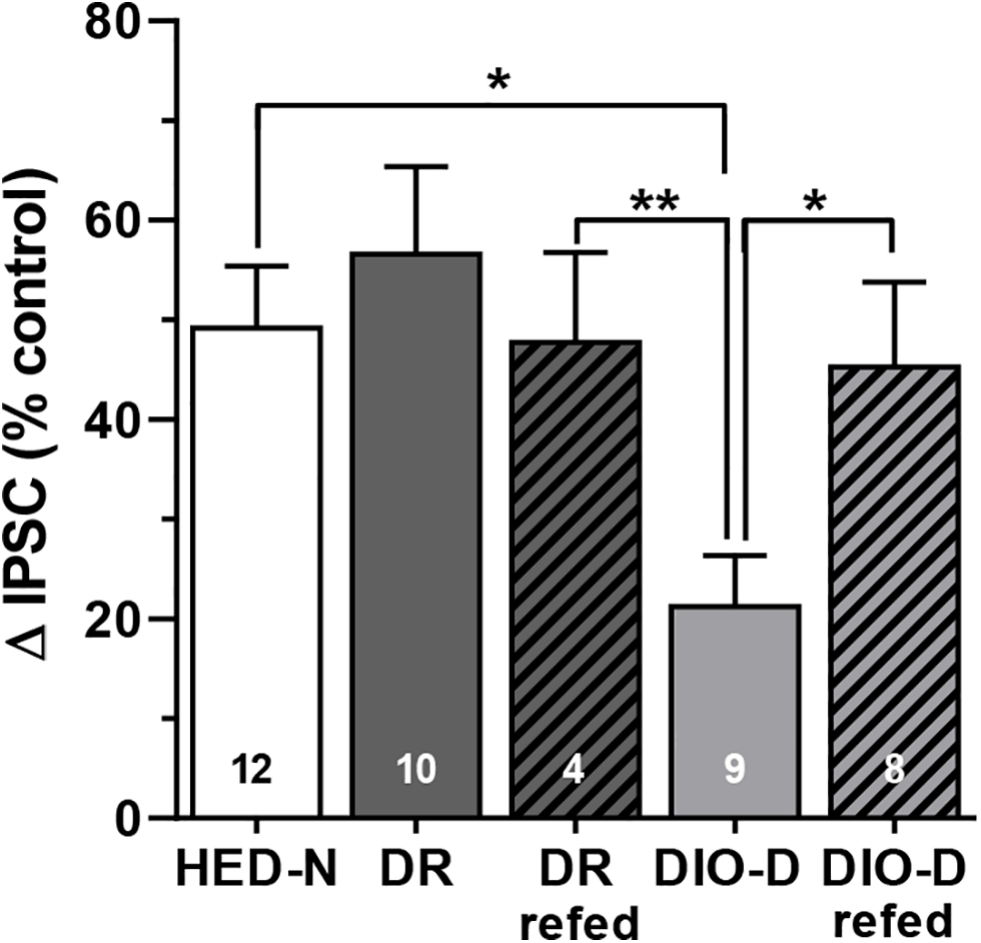
HED refeeding restores MTII effects in outbred DIO-D Sprague Dawley rats. Four weeks of HED refeeding (subsequent to ≥ 3 week chow challenge) restored NS neuron IPSC sensitivity to 10 nM MTII in DIO-D rats without affecting MTII responses in DR rats. Numbers of neurons studied are as indicated in each column. * p < 0.05; ** p < 0.01.

Our results indicated an attenuated anorexigenic drive in rats that defend a higher body weight setpoint even after cessation of HED feeding. To determine whether the loss of melanocortin sensitivity in the PVN of DIO-D animals is irreversible, we returned a subset of DR and DIO-D rats to the HED and determined if returning DIO-D animals to *ad libitum* HED would restore MTII responses in PVN. After 3–4 weeks of *ad libitum* HED-refeeding, IPSCs in NS neurons from DIO-D rats were more sensitive to MTII (DIO-D_refed_: +45.3 ± 8.5%, n = 9) than were those from age-matched DIO-D rats that remained on chow (DIO-D: +21.2 ± 5.2%, n = 9, p < 0.05; **Fig 7**). As a result, there were no longer any differences in MTII responses between DIO-D_refed_ rats and either age-matched HED-N rats or DR_refed_ rats (**Fig 7**). Therefore, although the DIO-D animals defend their body weight around a higher setpoint, presynaptic melanocortin responses in the PVN appear to retain their plasticity in response to changes in dietary energy density.

## Discussion

The homeostatic regulation of mammalian body weight must simultaneously accommodate normal growth until maturity and protect the organism against weight loss; this implies a unidirectional form of plasticity and a defense of a progressively higher body weight setpoint, at least until maturity. Our results suggest that a modest number of neurons in the PVN can regulate the elevated body weight setpoint. We show that in susceptible individuals, return to LED after prolonged HED exposure induces a loss of melanocortin sensitivity in a subset of the PVN neurons that would contribute to the defense of an elevated body weight setpoint, This loss is stable in DIO-D animals maintained on LED chow, but can be reversed by several weeks’ unfettered access to HED. Furthermore, this plasticity mirrors developmental changes in melanocortin sensitivity in the same inputs to the PVN we previously reported occurring during the postnatal period [27]. By contrast, the NPY responses diminish with increasing net weight. These presynaptic changes occur in a subset of PVN neurons which does not express postsynaptic responses to either NPY or to MC4R agonists.

Body weight setpoint integrates multiple, complex responses to external and intrinsic signals, but can be permanently altered in some individuals following prolonged exposure to HED. The PVN is an integral node in the hypothalamic network governing energy homeostasis [12, 38, 39]. Melanocortins and NPY acting in the PVN regulate food intake and appetite, which may at least in part result from regulating GABA release onto a subpopulation of medial parvocellular NS neurons [12, 13, 27]. To determine if this mechanism can be altered permanently by prolonged exposure to an energy-dense diet, we compared responses to MTII and NPY in mpPVN NS cells from HED-exposed and-naïve rats several weeks after all HED-fed animals were returned to chow. Our results demonstrate that NS neurons from rats that defend excess weight had reduced presynaptic responses to MTII, while the presynaptic responses to NPY did not differ from those in HED-N rats. This reduction in presynaptic sensitivity to the melanocortin agonist was induced specifically by HED exposure, both in inbred and outbred Sprague Dawley rats, and in individual animals was proportional to the magnitude of excess weight gained.

The central melanocortin system is a key component of long-term body weight regulation. Many prior studies indicate that this system is vulnerable to changes that result in derangements of energy balance in rodents and humans [14, 40]. The excitatory and inhibitory innervation of POMC neurons in the ARC is significantly rearranged both in inbred DIO rats and outbred mice fed HED [41], while postsynaptic MC4R activation is reduced 3-fold in mice maintained on HED [7]. Genetic disruption of central melanocortin signaling, including of PVN MC4Rs [16, 42], causes obesity in mice [43]. In humans, polymorphisms in melanocortin receptors are genetically linked with morbid obesity [44], while functional MC4R signaling is required for the beneficial effects of bariatric surgery [45]. The reduction in melanocortin sensitivity in the mpPVN of DIO-D rats is localized to specific presynaptic fibers originating at least in part from the DMN. MC4Rs are expressed in GABAergic DMN neurons [21, 22, 46], but act presynaptically in the PVN [13, 23, 27]. The *in situ* hybridization studies here showed that relative to DR rats, MC4R expression was selectively downregulated in the DMN, but not in the ARC or PVN of DIO-D rats. This is consistent with a reduction in presynaptic melanocortin signaling in GABA terminals arising from the DMN [21, 22, 46]. These results suggest that a highly localized alteration of MC4R expression in DMN neurons innervating the PVN may underlie a component of the defense of elevated body weight setpoint in DIO-D animals.

The suppression in sensitivity of GABAergic mpPVN afferents to energy balance-related signals was limited to melanocortin responses. Unlike in the leptin receptor-deficient, chow-fed, fatty Zucker rat [26], NPY actions on GABA inputs to NS neurons of either inbred or outbred DIO-D Sprague-Dawley rats were insensitive to excess weight gain. However, prolonged HED exposure can clearly affect NPY signaling in other nuclei. Indeed, we observed that the usually robust NPY-mediated inhibition of VMN neurons was strongly suppressed in the DIO-D animals. The inhibition of glutamatergic VMN neurons by NPY is thought to reduce their excitation of ARC POMC neurons, thereby reducing anorexigenic melanocortin output [33, 47]. Attenuated NPY action in the VMN of DIO-D rats might therefore reflect a compensation for the reduced melanocortin signaling within the PVN. Interestingly, NPY responses in DR rats were greater in the PVN than in HED-N and DIO-D rats, even though the melanocortin responses remained unaffected in DR. While we have no explanation yet for this finding, it is unlikely to contribute to the defense of elevated body weight in DIO-D animals.

Although there are populations of neurons in the PVN that are intrinsically sensitive to NPY and melanocortin actions [18], for a number of reasons, the cells we studied here appear to represent a different population of neurons. First, none of the neurons studied here demonstrated postsynaptic responses either to NPY or MTII, consistent with earlier reports [13, 23, 26, 27]. This does not reflect an inability of our methods to reveal postsynaptic actions of neuropeptides [33, 48, 49]. Second, from the position of the neurons we report here, in the middle of the PVN, we believe would have been very likely to encounter at least some MC4R/NPY-sensitive neurons, given the broad distribution within the PVN reported by Ghamari-Langroudi et al. [18]. As we did not observe any such postsynaptic responses, the selection criteria (mpPVN parvocellular neuron, tonically firing cell, prominent GABA responses [13, 23, 26, 27] may instead tend to exclude neurons with postsynaptic responses, despite the fact that about 50% of those cells were reported to express CRH, [27] as did many in the same region of the PVN in MC4R-GFP mice [18]. Intracellular recordings from PVN neurons retrogradely labelled from the rostral ventrolateral medulla in obese and lean Zucker rats demonstrated postsynaptic responses to MTII while no effects whatever of the melanocortin were observed on synaptic transmission [50], demonstrating the existence of neurons in rat PVN similar to those described by [18]. Interestingly the melanocortin responses were significantly greater in PVN neurons from obese than from lean Zucker rats, suggesting a physiological compensation for excess adiposity, as was observed in presynaptic NPY responses in PVN of obese Zucker rats [26]. While it is not known what the response of the MC4R-sensitive neurons in mouse PVN are to obesity, it appears certain that the neurons we have studied represent a different and probably nonoverlapping population of PVN neurons from those expressing postsynaptic melanocortin responses in mice.

Our results differ from those reported in experiments in which a global MC4R knockout was systematically restored in cre-expressing glutamatergic (*Vglut2*) or GABAergic (*Vgat*) neurons [51]. In these experiments, restoration of MC4R expression in glutamatergic neurons was sufficient to rescue normal body weight in these otherwise obese knockout mice, while restoration of MC4R expression in GABAergic neurons produced minimal effects on body weight changes, suggesting that, in mouse at least, MC4Rs on GABAergic neurons are not key in regulating body weight. This may imply differences in the expression of MC4Rs in neurons of mice vs. rats, or different roles for subpopulations of MC4R-expressing GABAergic neurons in mice. In any case, the alterations in MC4R expression in DMH seen in DIO-D but not DR animals are consistent with our hypothesis that GABAergic neurons are indeed involved in changes in body weight setpoint in rats, which more closely conform to human diet-related obesity than do mice.

The NPY and melanocortin systems reciprocally regulate feeding, energy expenditure and body weight. Loss of PVN melanocortin responses in DIO-D rats implies that NPY effects are relatively unopposed in that nucleus, which would result in the net accumulation of energy stores [12]. This imbalance would permit DIO-D animals to defend their elevated body weight despite eating a low energy diet. Indeed, the relationship between net weight gain and the MTII response was linear in all HED-exposed animals, while no such relationship with body weight existed among HED-N animals. While we cannot rule out that DIO-D animals reduced their energy expenditure to help defend the higher body weight, it is evident that they consumed the same number of calories on chow as they did earlier on HED. When DIO-D animals were refed with HED for 4–5 weeks, this restored PVN melanocortin sensitivity. This suggested that their melanocortin systems retain the ability to up- and downregulate around their elevated body weight setpoint in response to longer-term changes in dietary energy density. The plastic nature of this response is consistent with a body weight setpoint mechanism. Meanwhile, despite the considerable range of body weights seen in HED-N rats, neither their melanocortin nor NPY responses were correlated to body weight, suggesting that melanocortin system plasticity is restricted to conditions under which body weight setpoint is challenged.
